# Research on the dynamic viseme of the lip shape based on facial motion capture technology

**DOI:** 10.3389/fnbot.2022.922756

**Published:** 2022-09-26

**Authors:** Shengyin Zhu

**Affiliations:** College of Chinese Language and Literature, Northwest Normal University, Lanzhou, China

**Keywords:** facial motion capture, lip shape, viseme feature, articulatory, facial parameter feature

## Abstract

In the study of articulatory phonetics, lip shape and tongue position is the focus of linguists. In order to reveal the physiological characteristics of the lip shape during pronunciation, the author takes the Tibetan Xiahe dialect as the research object and defines the facial parameter feature points of the speaker according to the MPEG-4 international standard. Most importantly, the author uses the facial motion capture technology to obtain the dynamic lip viseme feature data, during the stop's forming-block, continuing-block, removing-block, and co-articulation with vowels in the CV structure. Through research and analysis, it is found that the distribution of lip shape change the characteristics of different parts' pronunciation is different during the stop's forming block. In the co-articulation with [a], the reverse effect is greater than the forward effect, which is consistent with the relevant conclusions in many languages obtained by many scholars through other experimental methods. The study also found that in the process of pronunciation, the movement of the lip physiological characteristics of each speaker is random to a certain extent, but when different speakers pronounce the same sound, they can always maintain the consistency of the changing trend of the lip shape characteristics.

## Introduction

Viseme refers to the physical state of the visible parts such as lips, tongue position, teeth, and jaw that correspond to phonemes in the process of pronunciation. A phoneme is the smallest phonetic unit that distinguishes meaning in a language, and the viseme corresponding to a phoneme is the smallest unit that distinguishes meaning in speech. For the vocal organs that are not visible in their natural state, the motion track can be extracted by means of ultrasound, and the extraction method of the lip motion track can be extracted by ultrasound, or by a camera or a motion capture device. Early researchers used ordinary cameras to capture data such as the mouth opening and lip width of speakers. Yao et al. (2000) photographed the lip shape change of the speaker with a camera, and described the internal and external contour of the lips by using the difference of the skin color of the lips. With the development of motion capture technology; more and more people apply this technology to linguistic research because it can accurately measure the coordinates of moving objects in three-dimensional space. In this article, the three-dimensional coordinate value of the lip shape of the speaker is collected through the face motion capture device.

Wang of Tsinghua University used front and side cameras to collect static viseme of speakers (Wang and Cai, 2003). Deng used Vicon Motion capture to collect facial motion data to study English viseme. Pan (2011) used infrared capture technology to collect lip viseme in Mandarin Chinese for speech perception research. Suo (2011) extracted the motion features of virtual human based on motion capture. He (2016) used infrared motion capture equipment to collect three-dimensional viseme features of the Tibetan Lhasa dialect. Shengyin (2020) used a helmet-mounted facial motion capture device to collect the lip features of speakers speaking the Tibetan Amdo dialect.

Although the above methods can obtain the corresponding data, the accuracy of the data is not high. Pan, He, and others use infrared technology to capture the lip movement track, but a few amounts of data will be lost when dealing with the relationship between objects and space. Due to the change of facial motion, sometimes adjacent feature points will overlap, so the computer will mistakenly consider it as one feature point. In this paper, on the basis of the research with the helmet-mounted device conducted by Zhu, the extraction method and data analysis process of speakers' viseme features is further optimized with the help of facial motion capture technology.

## Lip shape parameter setting

### Collection equipment

**Figure 1:**
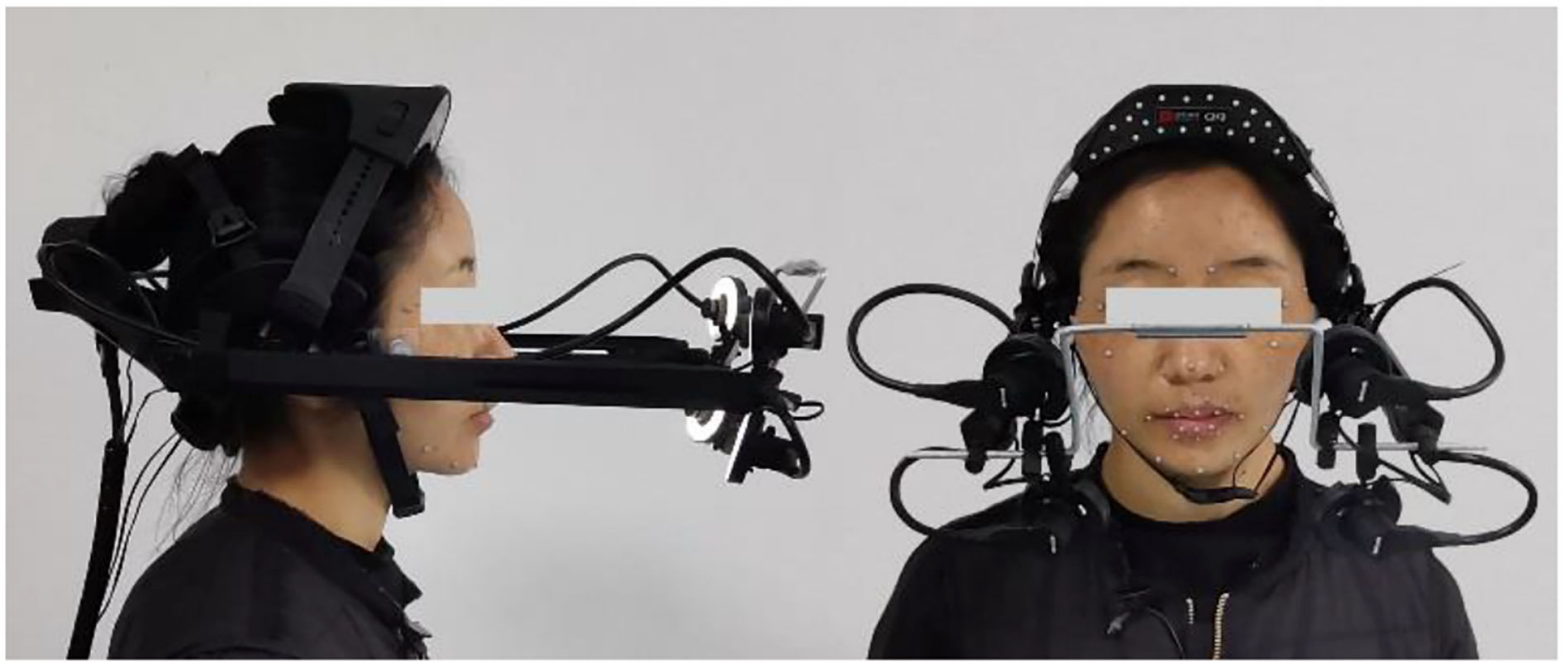
Collect the dynamic data of lip shape of the speaker lmc.

The device used for this collection is Vicon Cara, the world's first out-of-the-box facial expression capture system. At present, this device is mostly used for facial motion capture in animated movies, and the author of this article first applied this device to linguistic research in 2020. The advantage of this device is that it has 4 independent high-definition cameras, dedicated tracking computing software, and a data recorder, which can capture the full face at a larger angle. The subtle differences between lip movements can also be captured by high-resolution high-speed cameras. This equipment is a helmet-mounted instrument, which can be fixed on the same rigid body as the head, effectively avoiding the data error caused by the unconscious movement of the speaker's head. Also, it is easy to carry, does not need a professional site, and greatly facilitates the fieldwork for language surveys. The accuracy and convenience of data acquisition, it is better than the traditional acquisition method, as shown in Figure 1.

### Parameter definition

**Figure 2:**
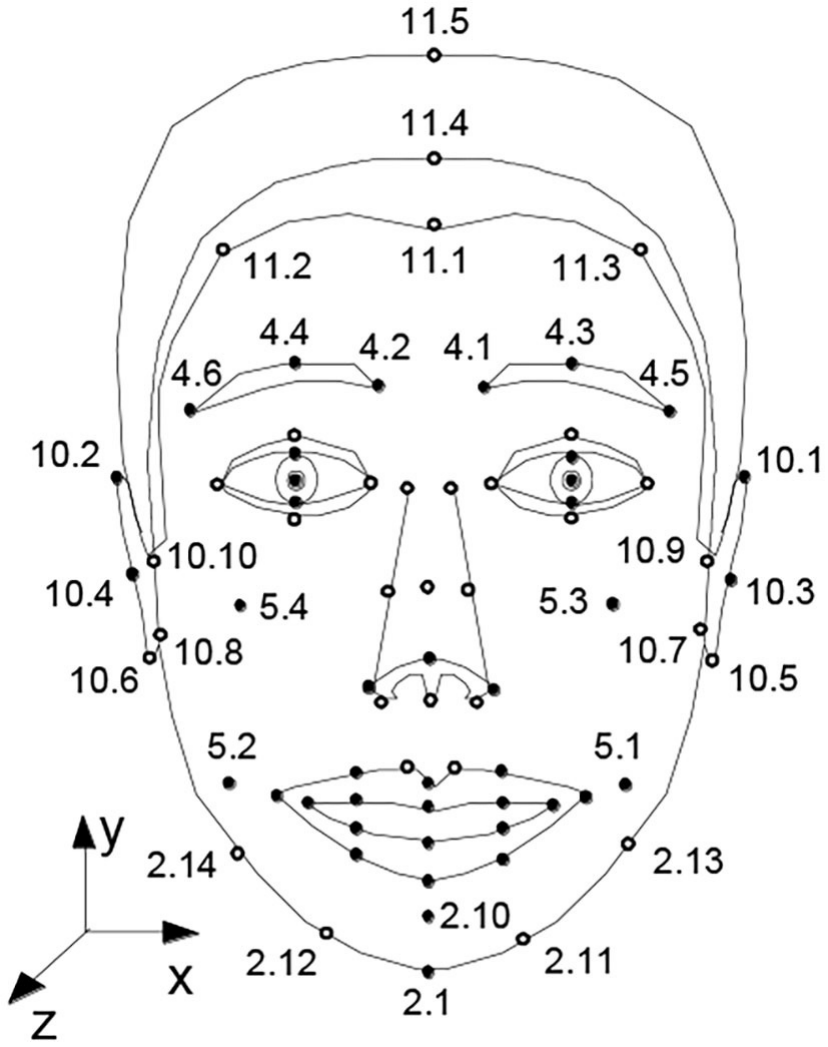
The FDP defined by the MPEG-4(Standard 2001).

The experiment in this paper defines the Facial Definition Parameter (FDP) according to the Moving Picture Experts Group (MPEG-4) standard. FDP has a total of 84 facial feature points, as shown in Figure 2. An Dandan (2009) uses 28 parameters to control a shape of the mouth according to the MPEG-4 standard. Considering the scope of research in this paper, only 36 points of lip shapes, mandibles, and auxiliary are selected. Among them, the lip shape is 16 points, and the other auxiliary points are 20.

Table 1 is the distribution of FDP and the description of feature points. There are 16 lip feature points, including inner lip and outer lip.

**Table 1:** The description of FDP.

**Articulation**	**Feature**	**Description**	**Articulation**	**Feature**	**Description**
**places**	**point**		**places**	**point**	
Chin	2.1	The middle point of the chin	Outer lip	8.1	The middle point of upper outer lip
	2.11	The right point of the chin		8.2	The middle point of lower outer lip
	2.12	The left point the chin		8.3	The right corner point of the outer lip
Inner lip	2.2	The middle point of the upper inner lip		8.4	The left corner point of the outer lip
	2.3	The middle point of the lower inner lip		8.5	The point next to the right corner of the upper outer lip
	2.6	The right point of the upper inner lip		8.6	The point next to the left corner of the upper outer lip
	2.7	The left point of the upper inner lip		8.7	The point next to the right corner of the lower outer lip
	2.8	The right point of the lower inner lip		8.8	The point next to the left corner of the lower outer lip
	2.9	The left point of the lower inner lip		8.9	The left point next to the center of the upper outer lip
Cheekbones	5.3	The right point of the cheekbones		8.10	The right point next to the center of the upper outer lip
	5.4	The left point of the cheekbones			

### The parameter extraction of upper lip protrusion, inner and outer lip height, and outer lip width

**Figure 3:**
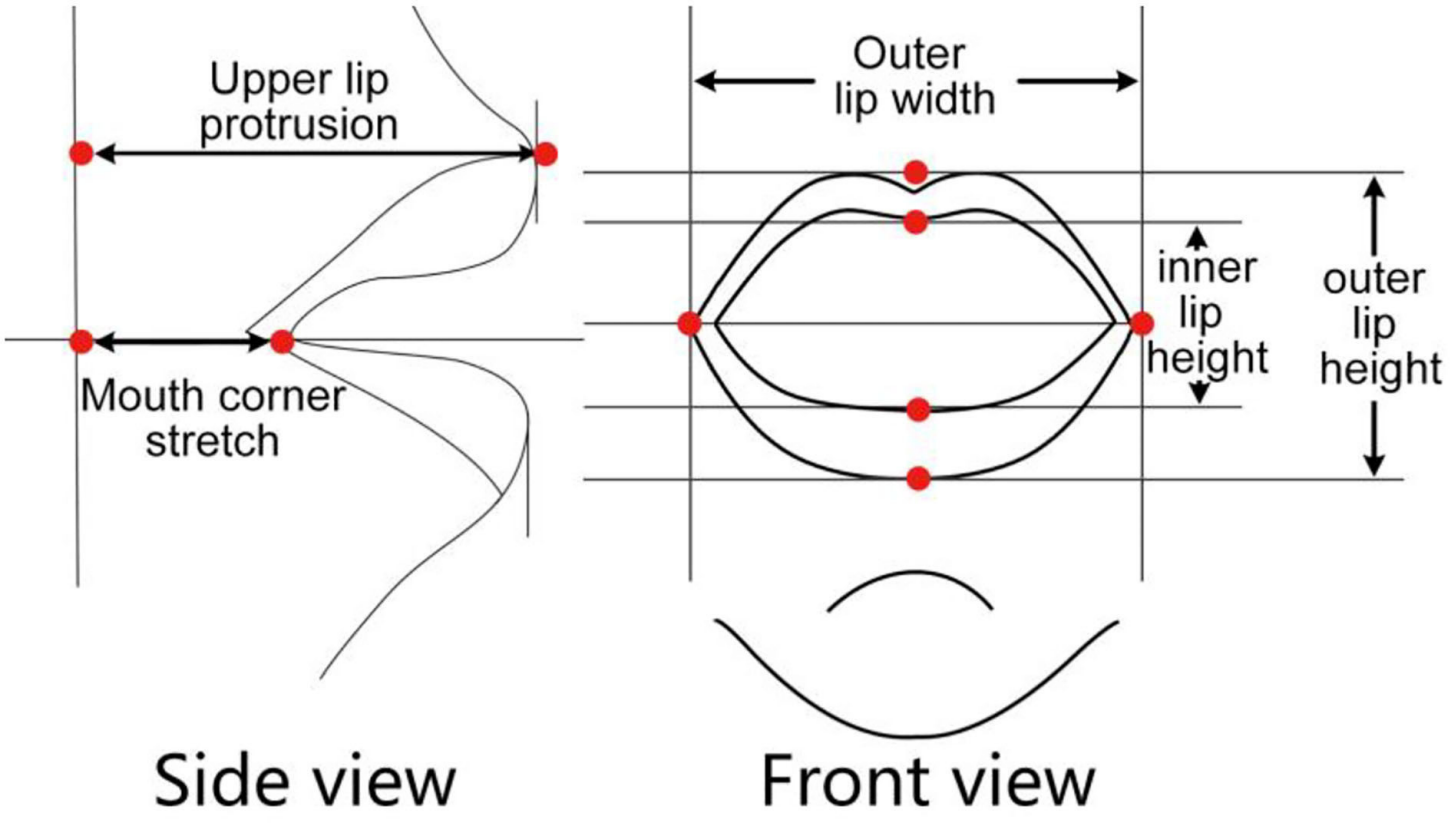
Parameter calibration.

Figure 3 is about the annotation of the extraction of each index data. The outer lip width and inner and outer lip height can be extracted directly, and the upper lip protrusion and the mouth corner stretch need to define a position to obtain the relative value. But when using this method, researchers must ensure that the feature point of the mouth corner stretch and the feature point of the upper lip protrusion is on the same rigid body.

### The parameter extraction of lower lip protrusion

In the process of pronunciation, the protrusion of the lower lip changes abundantly due to the influence of the mandible and the skin tissue. Therefore, the analysis of lip protrusion should be combined with the movement law and physiological structure of the mandible. During the pronunciation, the mandible is affected by the voice, and it will also move back and forth in addition to the rotation at the pivot point. And because of the influence of the physiological characteristics of different speakers, this change is not the same. Therefore, the displacement of the mandible and the independent speaker must be considered when calculating the protrusion of the lower lip. Wang (2000) and others used a fixed reference point on the side of the face, and the horizontal distance from the outer edge of the lower lip and the lip angle was defined as the lower lip protrusion; Wang and Cai (2003). After eliminating the rigid body motion of the head, the fixed The point is set as the reference point, and the horizontal distance between the reference point and the outer edge of the upper lip and the upper corner of the lower lip is defined as the protrusion of the upper lip and the protrusion of the lower lip;

Vatikiotis-Bateson and Ostry (1995) carried out a more detailed analysis of the movement of the mandible based on the physiological anatomy of the bones. It is believed that there are two kinds of movement of the mandible in the axial center rotation and forward and backward translation during the pronunciation; Based on the research of De Martino et al. (2006) simplified the movement of the mandible and put forward a method which used real-time sampling data of marked points on the mandible to calculate the approximate value of the rotation angle and translation distance of the condyle.

**Figure 4:**
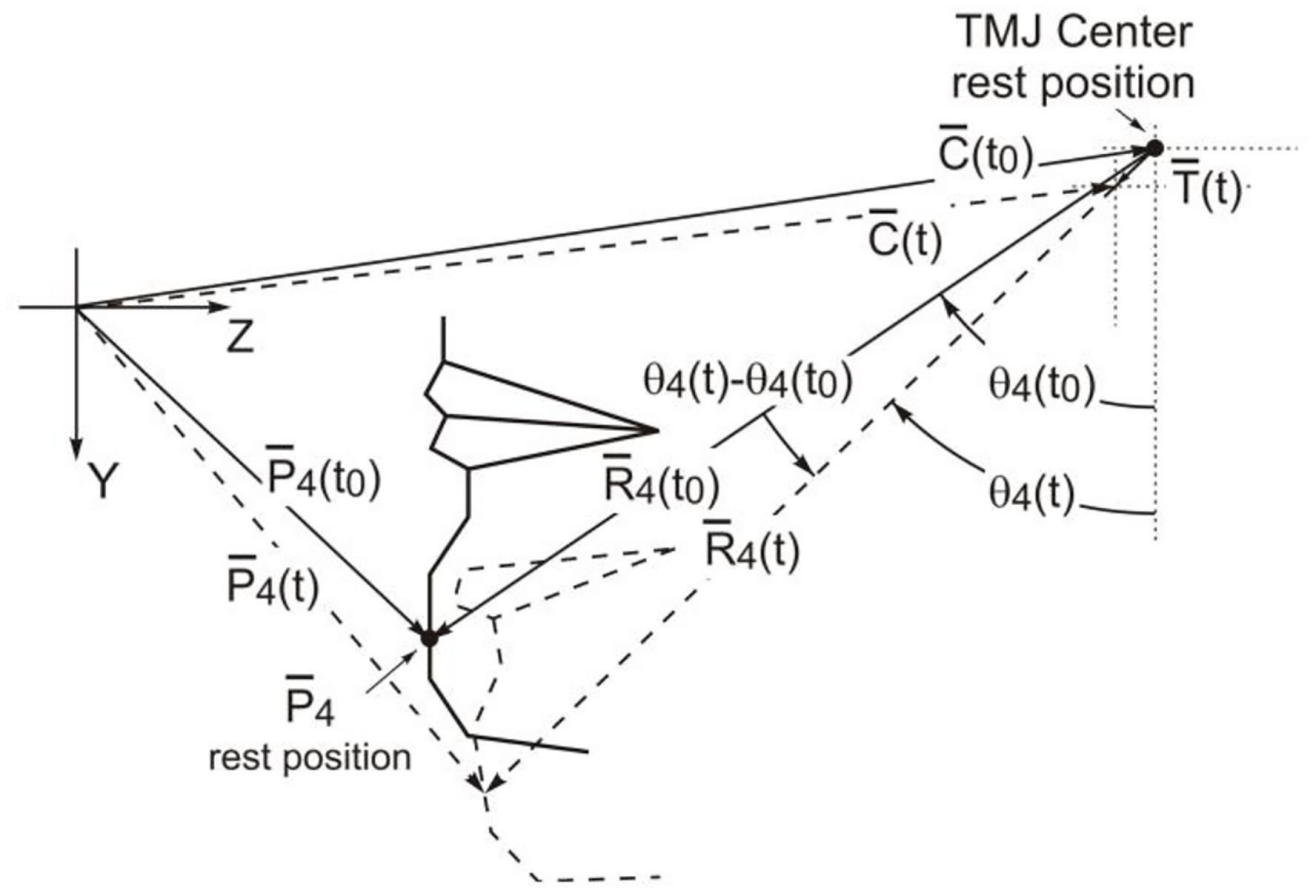
The condyle motion model defined by De Martino.

In Figure 4, De Martino set the sampling point P4 on the mandibular, mainly to solve the position change of the condyle during the pronunciation process, and used the translation amount to solve the change of the lower lip protrusion. The C(to) and C(t) represent the positions of the condyles, while the marked points starting with P represent the positions of the mandibular markers. First, the movement track of the condyle was converted from the marked point to the marked point for marking, and then the rotation movement was carried out with the marked point as the axis. Also, after completing the rotational movement of the mandibular opening, the current position is on the marked point and on the straight line where the three points lie. When the lips are closed, the position of the condyle is at the marked point. However, this is based on measurable positions.

**Figure 5:**
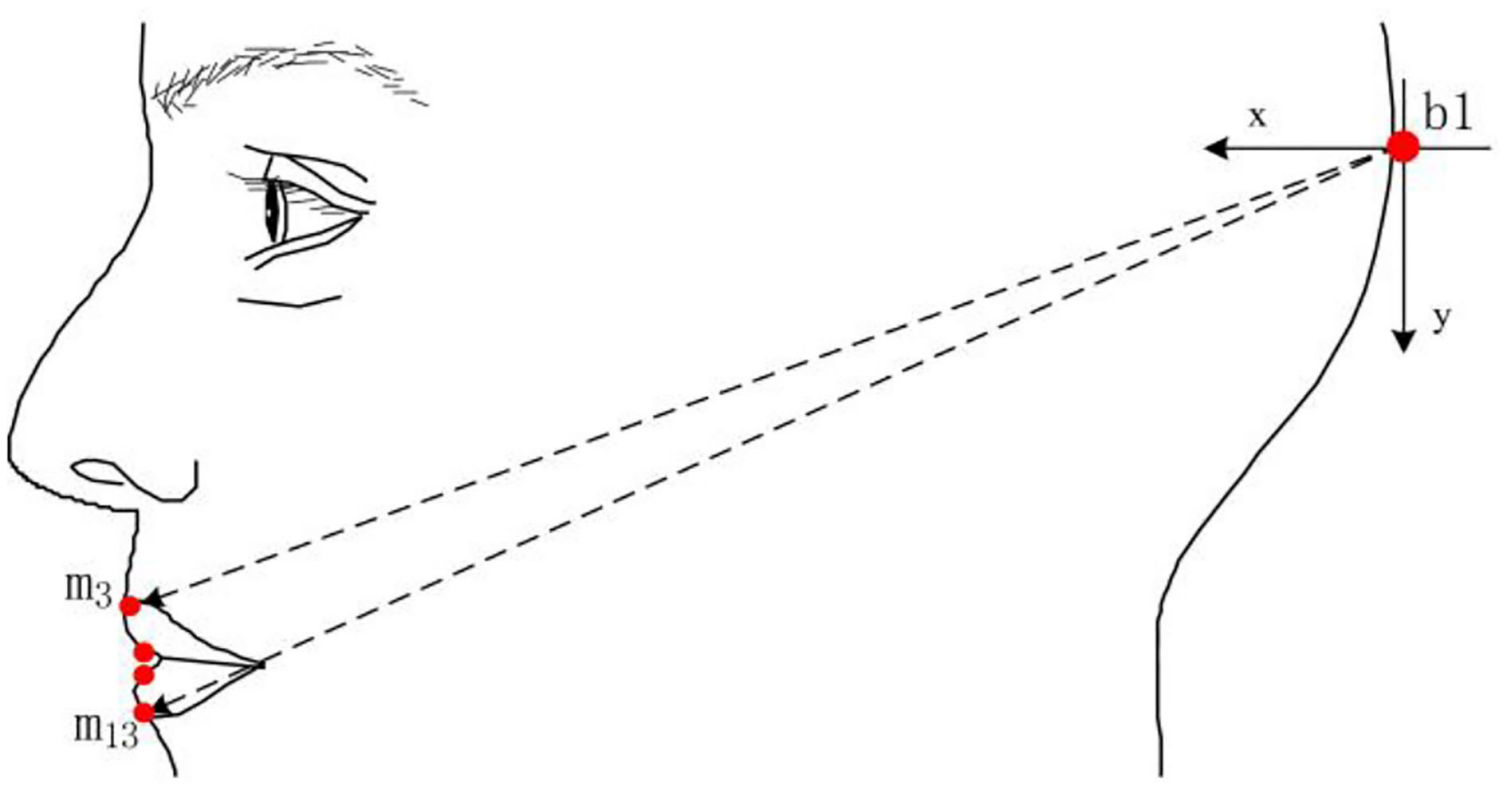
The marked points of the lip protrusion defined by He (2016).

De Martino's calculation of mandibular movement is feasible. It takes the rotation of the mandible into account but does not consider the translation of the mandible, and the data obtained is the relative value of the mandible. The main problems in the calculation process are as follows: in the collected data samples, the position of the condyle cannot be measured specifically; when the mandible is moved in translation, the marked points are on the same straight line. If the mandible is moved in rotational again during pronunciation, these three points are not necessarily on the same straight line, then the premise assumption mentioned above does not exist, and therefore accurate results cannot be obtained. When He (2016) collected the viseme in the Tibetan Lhasa dialect, on the basis of De Martino, he improved the mandibular and condyle movement model with the condyle as the axis. Although the mandible is generally rotated around the condyle as the axis, or it produces translational motion by itself, the accuracy of the data has been improved a lot. As is shown in Figure 5.

Compared with the research methods of different scholars, He's method of solving the protrusion of the lower lip is relatively complete. Therefore, the changes in the protrusion of the lower lip in this article can be directly extracted after partial optimization on this basis.

## Data processing

### Preprocessing of 3D dynamic viseme data

**Figure 6:**
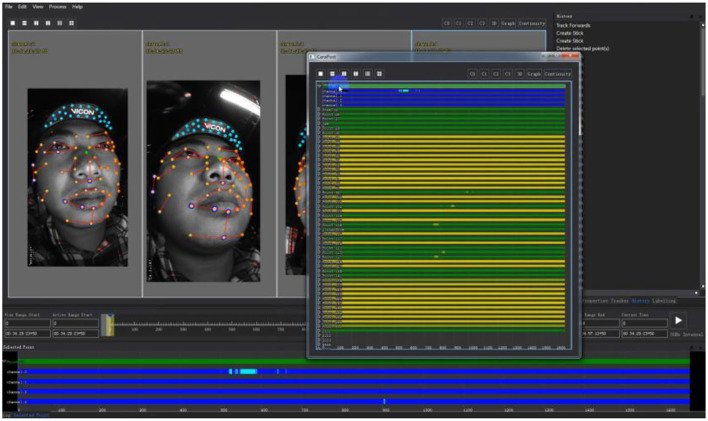
The process of data repair (The Image from Shengyin (2020)).

The preprocessing procedure of viseme data is as follows:

1. Import calibrationinformation for text matching: Import the video files recorded by Cara Live into Cara Post, check whether the signals of the four video channels meet the requirements, and check whether the facial marks are clearly visible according to the screen display.
2. Construct a three-dimensional environment by establishing seed points: First, establish a three-dimensional environment in Cara Post by selecting seed points and correlating points at different positions on the face. Then adjust the x, y, and z coordinate values to determine the position of the camera in the three-dimensional environment.
3. Construct the spatial position of the human face: Construct the three-dimensional point space coordinates of the human face and then check and calibrate.
4. Face topology: Face topology refers to selecting two marked points in a three-dimensional space to connect, and drawing a basic face structure diagram, which is very helpful for future observations. When drawing the face structure, connect adjacent marked points, which can be two points or multiple points, but it is not necessary to connect all the points together.
5. Name marked points: Naming marked points is to rename the marked points pasted on the face so that the corresponding coordinates of these points can be accurately known in the later data analysis.
6. Track the marked point and repair data: After the data processing is completed, the damaged data of the four channels can be repaired by executing related commands to ensure that the data reaches the required value. The main reason for the data repair is that the sharpness of the marked points in the previous period is not high or the camera position or focal length is not accurate.
7. Output the data: After the data recovery is completed, the correlation between the points is relatively fixed to ensure the stability of the data. As is shown in Figure 6, the blue bar indicates that each channel has data, and the green one indicates that only three channels have data. The yellow bar means that only two channels have data. If it red one appears, it means that only one channel has data or no data. If there is periodic data loss, just repair it. If all the information on the spot is lost, it needs to be collected again.

### The method of extracting dynamic viseme feature data

When the task of collecting the viseme data, a ^*^.bvh file will be got, which is the three-dimensional coordinate data of facial feature points. However, it is impossible to directly extract the three-dimensional coordinates of each point in the ^*^.bvh file, so only through a series of tedious conversion processes can the final required data be obtained.

1. Export the file in ^*^.bvh format through the file preprocessed by Cara Post.
2. After executing the ^*^.bvh file with Motion Builder, output the file in ^*^.fbx format.
3. Use Python to execute the program of extracting data and get a ^*^.json file. Opening this file, the data needed for the experiment can be clearly seen.
4. inally through data filtering, the available data is extracted and saved in the Excel worksheet. The first column in the Excel table is defined as the number of frames, followed by the value of outer lip width, outer lip height, upper lip protrusion, and lower lip protrusion. In order to facilitate the application of later data, only 2 decimal places are reserved.

### Data matching

Cara can also collect synchronized audio and video files while collecting 3D data files, but the synchronized audio and video files cannot meet the requirements of later audio data analysis. Therefore, in this experiment, an independent professional camera is used to obtain audio and video data, and then the audio and video files obtained by the independent camera are set synchronously with the audio and video files collected by Cara on the computer, so as to obtain high-quality audio and video files. Then it can be completely matched with the 3D data collected by Cara.

## Data analysis

### Pronunciation data

This paper takes the Tibetan Xiahe dialect as the research object and takes the stop sound as an example. There are 6 stops in the Xiahe dialect: /p, t, ph, kh, th, k/ (Zhang, 2014). /p/ is an unaspirated voiceless stop, which is a bilabial sound according to the articulation places. /p^*h*^/ is an aspirated voiceless stop, which is a bilabial sound according to the articulation places. /t/ is an unaspirated voiceless consonant, which is an alveolar according to the articulation places. /t^*h*^/ is an aspirated voiceless consonant, and it is an alveolar according to the articulation places. /k/ is an unaspirated voiceless consonant, which is a velar according to the articulation places. /k^*h*^/ is an aspirated voiceless consonant, which is a velar according to the articulation places. /p^*h*^/, /t/, and/k/ are selected for the stop sound experiment, followed by the vowel /a/, forming the co-articulation of the CV structure.

Table 2 is the pronunciation vocabulary of the stop sound experiment. The words selected in this experiment are all disyllabic words, and the three sounds are selected according to different articulation places. The bilabial /p^*h*^/ combined with the vowel /a/ is /p^*h*^a/, which corresponds to “
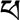
” (father) in Tibetan; the alveolar /t/ combined with the vowel /a/ and is /ta/, the corresponding Tibetan word is “
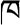
” (now); the tongue-root sound /k/ combined with the vowel /a/ is /ka/, and the corresponding Tibetan word is “
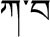
” (pillar). In order to facilitate the comparison and analysis of dynamic viseme data, each data is standardized, and the duration of the three sounds is controlled at 1.52 s (38 frames).

**Table 2:** Stops in Tibetan Xiahe dialect.

**No**.	**1**	**2**	**3**
stops	p^*h*^	t	k
Articulation places	Bilabial	Tongue lip	Tongue root
Tibetan	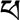	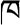	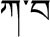
IPA	p^*h*^a	ta	ka
Duration(s)	1.52	1.52	1.52
Frames	38	38	38
Aspirated /unaspirated	Aspirated	Unaspirated	Unaspirated
Voiceless/voiced	Voiceless	Voiceless	Voiceless

The experimental data selects the viseme and the corresponding audio and video data of the speaker SJJ. The static viseme values of SJJ in the natural state are: outer lip width 5.9 cm, outer lip height 1.89 cm, upper lip protrusion 3.68 cm, lower lip protrusion 3.84 cm, and mouth corner stretch 2.25 cm. Tables 3–5 relatively show the dynamic data of the outer lip width, outer lip height, upper lip protrusion, lower lip protrusion, and mouth corner stretch of the sound “
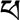
” [p^*h*^], “
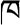
” [t], “
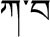
” [k] in the Tibetan Xiahe dialect. This paper takes the sounds p^*h*^a, ta and ka for example to analyze the lip shape dynamic viseme features.

**Table 3:** The dynamic data value of each part of “
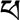
”/p^*h*^a/ in the Tibetan Xiahe dialect (unit: cm).

**Frame(25 frames per second)**	**Outer lip width**	**Outer lip height**	**Upper lip protrusion**	**Lower lip protrusion**	**Mouth corner stretch**
1	5.9	1.89	3.68	3.84	2.25
2	5.9	1.89	3.68	3.84	2.25
3	5.9	1.89	3.68	3.84	2.25
4	5.9	1.89	3.69	3.77	2.27
5	5.9	1.89	3.68	3.76	2.26
6	5.78	1.89	3.66	3.75	2.26
7	5.8	1.89	3.64	3.76	2.25
8	5.8	1.87	3.63	3.74	2.25
9	5.83	1.86	3.63	3.75	2.23
10	5.86	1.85	3.62	3.78	2.21
11	5.89	1.75	3.64	3.79	2.2
12	5.91	1.57	3.65	3.9	2.18
13	5.93	1.52	3.66	3.92	2.17
14	5.96	1.49	3.67	3.94	2.15
15	5.94	1.48	3.68	3.93	2.15
16	5.96	1.47	3.67	3.92	2.14
17	5.96	1.49	3.69	3.92	2.14
18	5.97	1.5	3.71	3.91	2.15
19	5.95	1.58	3.7	3.89	2.14
20	5.95	1.61	3.69	3.85	2.14
21	5.92	2	3.66	3.75	2.13
22	5.83	3.1	3.7	3.73	2.15
23	5.63	3.51	3.61	3.69	2.1
24	5.6	3.61	3.6	3.7	2.11
25	5.57	3.45	3.61	3.74	2.13
26	5.61	3.2	3.61	3.78	2.13
27	5.63	2.96	3.62	3.78	2.13
28	5.65	2.85	3.63	3.78	2.14
29	5.69	2.75	3.62	3.78	2.15
30	5.7	2.66	3.63	3.78	2.17
31	5.72	2.59	3.64	3.77	2.16
32	5.74	2.51	3.65	3.77	2.18
33	5.76	2.2	3.65	3.79	2.18
34	5.79	2.16	3.66	3.79	2.19
35	5.83	2.1	3.68	3.82	2.2
36	5.9	1.95	3.68	3.83	2.25
37	5.9	1.89	3.68	3.84	2.25
38	5.9	1.89	3.68	3.84	2.25

**Table 4:** The dynamic data value of each part of “
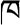
”/ta/ in the Tibetan Xiahe dialect (unit: cm).

**Frame(25 frames per second)**	**Outer lip width**	**Outer lip height**	**Upper lip protrusion**	**Lower lip protrusion**	**Mouth corner stretch**
1	5.9	1.89	3.68	3.84	2.25
2	5.9	1.89	3.68	3.84	2.25
3	5.9	1.89	3.68	3.84	2.25
4	5.9	1.89	3.68	3.84	2.25
5	5.9	1.92	3.68	3.84	2.25
6	5.86	1.97	3.7	3.8	2.3
7	5.85	1.95	3.71	3.81	2.29
8	5.84	1.92	3.7	3.81	2.29
9	5.86	1.89	3.72	3.83	2.28
10	5.87	1.9	3.72	3.81	2.29
11	5.87	1.9	3.73	3.81	2.3
12	5.84	1.97	3.71	3.78	2.29
13	5.82	2.1	3.67	3.7	2.27
14	5.8	2.29	3.61	3.6	2.24
15	5.73	2.79	3.64	3.63	2.2
16	5.7	2.91	3.63	3.62	2.19
17	5.73	2.7	3.64	3.68	2.16
18	5.74	2.64	3.63	3.69	2.17
19	5.66	2.89	3.64	3.63	2.08
20	5.6	3.27	3.62	3.57	2.01
21	5.61	3.44	3.59	3.56	1.98
22	5.61	3.48	3.61	3.6	1.99
23	5.58	3.42	3.61	3.62	2.01
24	5.58	3.4	3.62	3.62	2.01
25	6.6	3.32	3.63	3.63	2.02
26	5.61	3.18	3.63	3.64	2.03
27	5.65	2.98	3.64	3.66	2.06
28	5.67	2.76	3.64	3.68	2.09
29	5.74	2.46	3.65	3.7	2.16
30	5.79	2.28	3.66	3.71	2.2
31	5.82	2.26	3.65	3.71	2.22
32	5.9	2.23	3.68	3.75	2.2
33	5.92	2.2	3.68	3.75	2.22
34	5.98	1.95	3.68	3.76	2.22
35	5.9	1.89	3.68	3.79	2.25
36	5.9	1.89	3.68	3.83	2.25
37	5.9	1.89	3.68	3.84	2.25
38	5.9	1.89	3.68	3.84	2.25

**Table 5:** The dynamic data value of each part of “
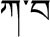
”/ka/ in Tibetan Xiahe dialect (unit: cm).

**Frame(25 frames per second)**	**Outer lip width**	**Outer lip height**	**Upper lip protrusion**	**Lower lip protrusion**	**Mouth corner stretch**
1	5.9	1.89	3.68	3.84	2.25
2	5.9	1.89	3.68	3.84	2.25
3	5.9	1.89	3.68	3.84	2.25
4	5.77	1.92	3.67	3.71	2.25
5	5.76	1.96	3.66	3.7	2.25
6	5.77	1.99	3.65	3.69	2.25
7	5.74	2.1	3.64	3.63	2.23
8	5.73	2.28	3.61	3.54	2.21
9	5.73	2.49	3.6	3.5	2.19
10	5.65	2.95	3.63	3.57	2.18
11	5.65	3.01	3.62	3.58	2.17
12	5.65	2.85	3.63	3.62	2.17
13	5.68	2.75	3.63	3.64	2.18
14	5.69	2.7	3.64	3.65	2.19
15	5.7	2.68	3.65	3.66	2.19
16	5.71	2.62	3.64	3.67	2.2
17	5.7	2.65	3.65	3.67	2.21
18	5.71	2.67	3.65	3.67	2.2
19	5.69	2.75	3.64	3.66	2.19
20	5.67	2.85	3.65	3.65	2.17
21	5.61	3.07	3.65	3.63	2.12
22	5.56	3.33	3.65	3.63	2.09
23	5.53	3.44	3.63	3.65	2.08
24	5.54	3.36	3.63	3.67	2.09
25	5.55	3.29	3.64	3.69	2.1
26	5.57	3.21	3.64	3.72	2.11
27	5.59	3.04	3.64	3.73	2.13
28	5.65	2.76	3.62	3.72	2.14
29	5.7	2.52	3.6	3.72	2.16
30	5.73	2.38	3.61	3.71	2.18
31	5.79	2.15	3.6	3.69	2.2
32	5.81	2.06	3.61	3.7	2.19
33	5.84	1.95	3.62	3.72	2.19
34	5.87	1.89	3.65	3.75	2.22
35	5.9	1.89	3.66	3.79	2.23
36	5.9	1.89	3.68	3.84	2.25
37	5.9	1.89	3.68	3.84	2.25
38	5.9	1.89	3.68	3.84	2.25

### Acoustic analysis

**Figure 7:**
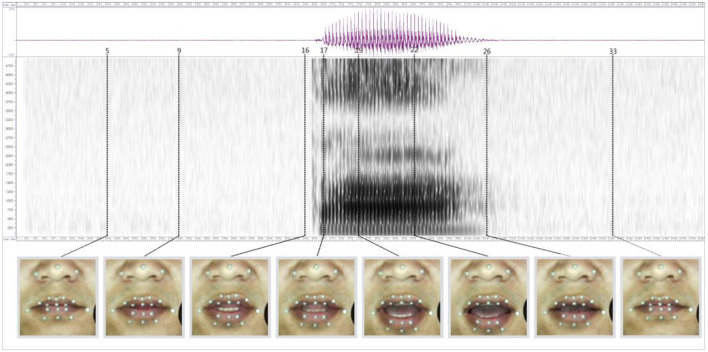
The sonogram, spectrogram, and lip shape viseme of the target position of the Tibetan “
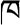
”.

Figure 7 is the sound wave map, spectrogram, and lip shape viseme of the target position of the Tibetan sound “
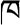
”. When the sound /ta/ is pronounced, the stop /t/ and the vowel /a/ form a double phoneme CV structure. The audio length is 15.20 ms, and the corresponding viseme sequence diagram has a total of 38 frames. The amplitude shown in the sonogram is between 2,753 and −1,397 Hz. It can be seen in the spectrogram that there is a blocking segment of consonants before the 16th frame, and there is no obvious random pattern. In the vicinity of frames 16–17, there is a relatively obvious amplitude and a short duration. This is the spike of the stop /t/. There is no obvious random pattern on the spike which is a way to judge the character of the unaspirated stop. Behind the 19th frame is the audio signal of the vowel /a/. According to the data distribution of the formant, F1 is 760 Hz, F2 is 1,220 Hz, and F3 is 2,400 Hz. The formant distribution is consistent with that of /a/ in the vowel experiment. In the acoustic analysis of /t/, 8 target viseme maps of the speaker are selected for comparative analysis, which are the 5th, 9th, 16th, 17th, 19th, 22th, 26th, and 33rd frames respectively. Judging from the static viseme map, the first 5 frames of lip shape remain unchanged in the preparation stage and frames 4–16 are the blocking segments of consonants. Judging from the change of the target viseme, the lips are slightly open. From the target viseme map of frames 16 and 17, it can be seen that the tongue is against the palate, and the tongue suddenly leaves when it is blocked. In the target viseme map of frame 19, it can be seen that the tongue has left the palate at this time, so the airflow rushes out as the resistance-removing section. According to the change of lip shape from frame 19 to frame 22, the target position of the vowel /a/ has been reached.

### Dynamic viseme analysis

Static viseme refers to the state of the visual organ that is still at a certain time during pronunciation. However, researchers have found that when people pronounce, the viseme is a continuous dynamic process, so a single static picture cannot completely describe the pronunciation characteristics. Dynamic viseme refers to a process of recording people's pronunciation, which can accurately reflect the pronunciation rules and visual habits. At the same time, it records the continuous and natural change process of viseme, which makes the recorded viseme characteristic data more accurate, and is also conducive to the study of phonetics.

**Figure 8:**
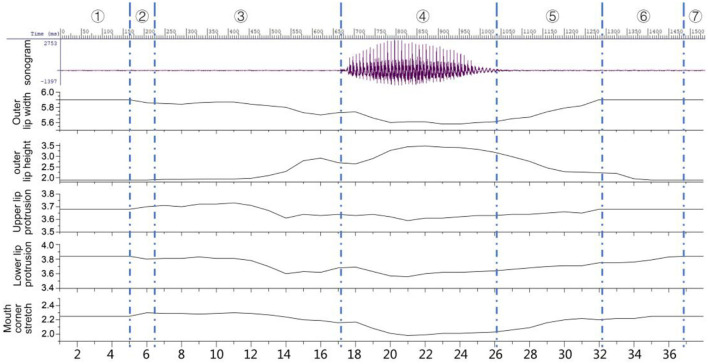
The sonogram and parameter change of lip shape of Tibetan “
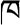
”.

Figure 8 is the sonogram of the Tibetan “
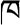
” and the curve graph of the outer lip width, the outer lip height, the upper and lower lip protrusion, and the mouth corner stretch.

The first line is the sonogram, whose horizontal axis is milliseconds and the vertical axis is the amplitude of speech. The second is the width of the outer lip, the third is the height of the outer lip, the fourth is the protrusion of the upper lip, the fifth is the protrusion of the lower lip, and the sixth is the stretch of the mouth corners. The horizontal axis of the second to sixth lines is the frame, and the vertical axis is cm. The whole pronunciation process can be divided into 7 parts, of which 1, 2, 3, 5, 6, and 7 are silent and 4 are vocal. 1, 2 and 3 parts are the preparation for pronunciation, and parts 4, 5, and 6 are the end of pronunciation. Parts 1 and 7 are in a natural state, and all mouth shape parameters have no change. “Duration of vocalization” refers to the part with sound in the whole pronunciation process, which is the focus of pure acoustic research, but “the period of silence” is more important than the duration of vocalization in the research of visual speech in a sense. For example, when consonants are pronounced, the blocking stage is silent, but the change of lip shape is more complex from the natural state to the end of the blocking. So the duration of vocalization and the period of silence are the important indexes of this paper.

The specific analysis is as follows:

1. Part 1 and part 7 are in the state of preparation, and there is no change in the mouth shape.
2. Part 2 is in the silent state, and all the indicators begin to change except the height of the outer lip. When the sound /t/ is pronounced, the tip of the tongue needs to touch the upper gums to block the airflow, and the lower jaw moves slightly backward. Therefore, the width of the outer lip is narrowed to 5.85 cm, and the protrusion of the upper lip is obvious while the lower lip contracts. Near the 6th frame, the protrusion of the upper lip is 3.7 cm higher than the natural state value, and the protrusion of the lower lip is 3.8 cm lower than the natural value. The stretch of the mouth corner moves forward to 2.3 cm around 6 frames due to the forward movement of the tongue. Judging from the change of the curve, in part 2, all the articulation places have been prepared for the blocking.
3. Part 3 is also in the silent state, and all the articulation places are involved in the pronunciation. This part is the preparation section of each articulation placed before pronunciation, and it is also the blocking section of the stop /t/. Since the blocking has been done in part 2, in this part, the changes mainly concentrated on the late stage of blocking. From the graph, between 6 and 12 frames, each articulation place is kept for a period of time within a small variation range. The values around 10 frames are: the outer lip width is 5.87 cm, the outer lip height is 1.9 cm, the upper and lower lip protrusions are 3.72 and 3.81 cm, respectively, and the mouth corner stretch is 2.29 cm. In frames 13–17, the airflow in the oral cavity has been gathered, and the parameters of different articulation places have changed greatly. The width of the outer lip is narrowed, the height of the outer lip is increased, and the tongue is ready to leave the upper soft palate.
4. Part 4 is in the vocal state, and all the articulation places are involved in the pronunciation. The changes in the lip shape are related to the nature of the voice. This part is the process of changing the lip shape of the stop /t/ into that of the vowel /a/, and the changes in each part of the lip shape are more complicated. Around frame 17, the tip of the tongue leaves the hard palate quickly, and an anterior airflow can be seen on the spectrogram. After the blocking is removed, the airflow in the mouth rushes out and bursts into a sound. At this time, the lips relax and open naturally. The width of the outer lip near the 18th frame is 5.74 cm, the height of the outer lip is 2.64 cm, and the protrusions of the inner and outer lips are 3.63 cm and 3.64 cm, respectively, and the stretch of the corner of the mouth is 2.17 cm. After removing the blocking, the static target viseme value of vowel /a/ of each articulation place is reached in the vicinity of 21 frames. The width of the outer lip was 5.61 cm, the height of the outer lip was 3.44 cm, and the protrusions of the upper and lower lips were 3.59 and 3.56 cm, respectively. The stretch of the mouth corners is 1.98 cm. Combined with the target viseme value of /a/ when the sound /p^*h*^/ is pronounced, it is found that when the sound /ph/ is pronounced, the static target viseme value of /a/ appears around 24 frames, and the outer lip width is 5.6 cm, the outer lip height is 3.61 cm, the upper and lower lip protrusions are 3.6 and 3.7 cm, respectively, and the stretch of the corners of the mouth is 2.11 cm. The change values of the two sounds are not very different, and the vowel /a/ is similarly affected by the plosive bilabial /p^*h*^/ and the alveolar /t/.
5. Parts 5 and 6 are in the silent state, and all the articulation places are involved in the pronunciation. This part is the process of restoring each articulation place to its natural state after the end of pronunciation. All articulation places involved in pronunciation are reset. The vocalized segment accounts for a little more than 1/3 of the whole process of pronunciation. Pan (2011) believes that the duration of vocalization only accounts for half of the whole process of pronunciation in the study of Chinese Putonghua viseme.

With the facial motion capture technology, the dynamic lip viseme feature data are obtained during the stop's forming-block, continuing-block, removing-block, and co-articulation with vowels in the CV structure. The results show that when the double lip [p ^*h*^]is pronounced during the forming - block, the lips closed tightly, except for the lip height, the change of each part was small, and the co-articulation process with vowels after removing the block changed greatly. The changes in the viseme index of the sound [t] are concentrated in the early stage of forming-block, and the sound [k] is concentrated in the late stage of forming-block, indicating that the different positions of articulation will directly affect the distribution of lip shape changes during forming-block. In addition, the data of the co-articulation process of the three sounds and the low vowel [a] after removing-block the show that the reverse effect of the co-articulation is greater when it is pronounced with [a] in the CV structure, which is consistent with the relevant conclusions in many languages obtained by many scholars through other experimental methods.

## Conclusion

The research methods of lip shape viseme are also different for different purposes, which also provides new perspectives and rich materials for verifying and further exploring the physiological characteristics of lip shape viseme. Existing studies mainly focus on the static relationship between lip shape viseme and phonemes, which provides important inspiration for determining the dynamic relationship between lip shape viseme and phonemes. However, it is far from enough to fully understand the physiological mechanism of lip shape in a language. The current research still has problems such as imperfect research methods and limited research angles. Therefore, in future research, on the basis of the relationship between lip shape viseme and phoneme, with motion capture, the dynamic viseme feature of lip shape will be further explored, and also combined with research evidence in other fields to make a more comprehensive analysis of the lip shape feature of articulatory phonetics.

This paper is the phased achievement of the Gansu provincial social science planning project “Research on Lip Shape Dynamic Viseme of Vowels in Tibetan Xiahe Dialect and the Database Construction” (2021YB045). It is also supported by the project “Research on Dynamic Viseme of Vowels in Tibetan Xiahe Dialect” for the improvement of young teachers' scientific research ability at Northwest Normal University.

## Data availability statement

The original contributions presented in the study are included in the article/supplementary material, further inquiries can be directed to the corresponding author.

## Ethics statement

The studies involving human participants were reviewed and approved by the College of Chinese Language and Literature of Northwest Normal University. The patients/participants provided their written informed consent to participate in this study. Written informed consent was obtained from the individual(s) for the publication of any potentially identifiable images or data included in this article.

## Author contributions

The author SZ is responsible for all the work concerning the study.

## Funding

This paper is the phased achievement of the Gansu provincial social science planning project Research on Lip Shape Dynamic Viseme of Vowels in Tibetan Xiahe Dialect and the Database Construction (2021YB045). It is also supported by the project Research on Dynamic Viseme of Vowels in Tibetan Xiahe Dialect for the improvement of young teachers' scientific research ability in Northwest Normal University.

## Conflict of interest

The author declares that the research was conducted in the absence of any commercial or financial relationships that could be construed as a potential conflict of interest.

## Publisher's note

All claims expressed in this article are solely those of the authors and do not necessarily represent those of their affiliated organizations, or those of the publisher, the editors and the reviewers. Any product that may be evaluated in this article, or claim that may be made by its manufacturer, is not guaranteed or endorsed by the publisher.
